# CRISPR-Cas9 multiplex genome editing of the hydroxyproline-*O-*galactosyltransferase gene family alters arabinogalactan-protein glycosylation and function in *Arabidopsis*

**DOI:** 10.1186/s12870-020-02791-9

**Published:** 2021-01-06

**Authors:** Yuan Zhang, Michael A. Held, Dasmeet Kaur, Allan M. Showalter

**Affiliations:** 1 Molecular and Cellular Biology Program, Ohio University, Athens, OH 45701–2979 USA; 2 Department of Environmental & Plant Biology, Ohio University, Athens, OH 45701–2979 USA; 3 Department of Chemistry & Biochemistry, Ohio University, Athens, OH 45701–2979 USA

**Keywords:** *Arabidopsis*, Arabinogalactan-proteins, CRISPR/Cas9, Hydroxyproline-rich glycoprotein, Hyp-*O*-galactosyltransferase, Pollen, Reproduction, Yariv, Root hair, Seed germination

## Abstract

**Background:**

Arabinogalactan-proteins (AGPs) are a class of hydroxyproline-rich proteins (HRGPs) that are heavily glycosylated (> 90%) with type II arabinogalactans (AGs). AGPs are implicated in various plant growth and development processes including cell expansion, somatic embryogenesis, root and stem growth, salt tolerance, hormone signaling, male and female gametophyte development, and defense. To date, eight Hyp-*O*-galactosyltransferases (GALT2–6, HPGT1–3) have been identified; these enzymes are responsible for adding the first sugar, galactose, onto AGPs. Due to gene redundancy among the *GALTs*, single or double *galt *genetic knockout mutants are often not sufficient to fully reveal their biological functions.

**Results:**

Here, we report the successful application of CRISPR-Cas9 gene editing/multiplexing technology to generate higher-order knockout mutants of five members of the *GALT* gene family (*GALT2–6*). AGPs analysis of higher-order *galt* mutants (*galt2 galt5*, *galt3 galt4 galt6*, and *galt2 galt3 galt4 galt5 gal6*) demonstrated significantly less glycosylated AGPs in rosette leaves, stems, and siliques compared to the corresponding wild-type organs. Monosaccharide composition analysis of AGPs isolated from rosette leaves revealed significant decreases in arabinose and galactose in all the higher-order *galt* mutants. Phenotypic analyses revealed that mutation of two or more *GALT* genes was able to overcome the growth inhibitory effect of β-D-Gal-Yariv reagent, which specifically binds to β-1,3-galactan backbones on AGPs. In addition, the *galt2 galt3 galt4 galt5 gal6* mutant exhibited reduced overall growth, impaired root growth, abnormal pollen, shorter siliques, and reduced seed set. Reciprocal crossing experiments demonstrated that *galt2 galt3 galt4 galt5 gal6* mutants had defects in the female gametophyte which were responsible for reduced seed set.

**Conclusions:**

Our CRISPR/Cas9 gene editing/multiplexing approach provides a simpler and faster way to generate higher-order mutants for functional characterization compared to conventional genetic crossing of T-DNA mutant lines. Higher-order *galt* mutants produced and characterized in this study provide insight into the relationship between sugar decorations and the various biological functions attributed to AGPs in plants.

**Supplementary Information:**

The online version contains supplementary material available at 10.1186/s12870-020-02791-9.

## Background

Arabinogalactan-proteins (AGPs) belong to the hydroxyproline-rich glycoprotein (HRGP) superfamily and are distinguished by their type II arabinogalactan (AG) polysaccharide chains which extensively decorate hydroxyproline (Hyp) residues in the protein backbones of AGPs. AGPs play critical roles at plant cell surfaces and function in plant growth, development, cellular signaling, wounding/defense, and programmed cell death [1]. AGPs typically contain dipeptide motifs such as Ala-Hyp, Ser-Hyp, Thr-Hyp, Val-Pro, Gly-Pro, Thr-Pro, and are glycosylated on noncontiguous Hyp residues. Addition of the various sugars to AGPs occurs in a stepwise fashion and requires the action of a large number of distinct enzymes, called glycosyltransferases (GTs) [2]. According to current models, AGPs consist of a backbone of galactosyl residues connected by β-1,3 linkages with branches of galactosyl and arabinosyl residues (type II AGs) that are β-1,6-linked; these side branches also contain other less abundant sugars such as β-(methyl) glucuronic acid (GlcA/MeGlcA), α-rhamnose (Rha), and α-fucose (Fuc) as terminal residues [3].

To date, eight Hyp-*O*-galactosyltransferases (GALT 2–6 and HPGT1–3) from the Carbohydrate Active Enzyme (CAZy) GT31 family were identified to function in catalyzing the addition of the first sugar, galactose, onto Hyp residues in AGPs of *Arabidopsis*. HPGT1–3 contain a GALT domain in their protein sequences, whereas GALT2–6 contain both a GALT domain and a GALECTIN domain [4]. Previously, our lab has characterized single and double mutants (*galt2, galt3, galt4, galt5, galt6,* and *galt2 galt5*) for the five *GALT* genes. All five GALTs demonstrated Hyp-*O*-galactosyltransferase (GALTs) activity in vitro [5, 6]. Single *galt* mutants exhibited less glycosylated (i.e., β-Gal-Yariv reagent precipitated) AGPs [6]. While the *hpgt1 hpgt2 hpgt3* triple mutant exhibited pleiotropic effects including a dwarf phenotype, longer root hairs, smaller rosettes, and shorter siliques, the corresponding *hpgt* single mutants displayed no distinct abnormal phenotypes [7]. Furthermore, both the *galt2 galt5* double mutant and *hpgt* triple mutant had ~ 40% and ~ 70% reductions in glycosylated AGPs in young seedlings, respectively [8].

Another β-1,3-galactosyltransferase named KNS4/UPEX1 from the GT31 family was recently characterized in *Arabidopsis*; this enzyme is thought to be involved with the synthesis of the β-1,3-galactan backbone [9]. Unlike other *GALT* and *HPGT* genes already mentioned, which are broadly expressed, the *KNS4/UPEX1* gene was only expressed in the inflorescence meristem. Mutant analyses found that KNS4 is critical for exine formation. The *kns4* mutants exhibited collapsed pollen, shorter siliques, and reduced fertility [9]. Two other GALTs, AtGALT29A and AtGALT31A were also discovered that function in attaching galactose onto β-1,6 galactan side chains. AtGALT29A and AtGALT31A when co-expressed, worked cooperatively by forming a protein complex. Also, single *galt31a* mutants exhibited abnormal cell division and an embryo lethal phenotype [10]. Two α-1,2-fucosyltransferases (FUTs), FUT4 and FUT6, from the GT37 family were shown to be responsible for transferring fucose to AGPs in *Arabidopsis* [11]. Loss of function of *FUT4* or *FUT6* resulted in a reduction of fucose and xylose on the AGPs, and a *fut4 fut6* double mutant contained no detectable fucose and were hypersensitive to salt stress [12, 13]. Additionally, another GT named RAY1, was identified as a putative β- arabinofuranosyltransferase for AGPs in *Arabidopsis*, despite the fact that only α-Ara*f* modified AGPs are reported to exist in nature [14]. Nonetheless, the *ray1* mutant showed a reduction of arabinofuranose (Ara*f*) residues in AGPs. This mutant also exhibited shorter root length along with a dwarf phenotype. It is noteworthy to mention that glucuronic acid is a relatively minor component and the only negatively charged sugar in AGPs. Recently, three glucuronosyltransferase (*GLCAT*) genes, *GLCAT14A*, *GLCAT14B*, and *GLCAT14C*, from the GT43 family were identified through co-expression analyses with AGPs in *Arabidopsis* [15]. Allelic T-DNA insertional mutants for *GLCAT14A*, named *glcat14a-1* and *glcat14a-2,* displayed a reduction in β-GLCAT activity, as well as ~ 12% increase in Gal and ~ 12% decrease in Ara content in AGPs compared to wild-type (WT) *Arabidopsis*. Both mutants also showed an ~ 30% increase in both root and hypocotyl lengths [15, 16]. Most recently, GLCAT14A and GLCAT14B were found redundantly regulating seed germination, root hair and trichome formation, seed mucilage formation, pollen shape, and silique development [17]. Both *glcat14a glcat14b* and *glcat14a glcat14b glcat14c* showed more glycosylated AGPs in rosette leaves, stems, and siliques [17]. Moreover, single, double, and triple *glcat *mutants demonstrated much less calcium binding on their AGPs compared to WT [17].

Given that AGPs generally consist of approximately 90% carbohydrate and 10% protein by weight, AG polysaccharides are believed to play a key role in AGP biological functions. However, as mentioned earlier, at least eight Hyp-*O*-GALTs from the GT31 family are known to be involved in initiating AGP glycosylation. Moreover, single *galt* mutants showed only subtle or no obvious phenotypes, and two higher-order *galt* mutants (*galt2 galt5* and *hpgt1 hpgt2 hpgt3*) only had partial reductions in the amount of glycosylated AGPs. This provides evidence that other *GALTs* are likely compensating when one or few *GALT* genes are non-functional and indicates gene redundancy exists within this family in *Arabidopsis* [5–8]. Clearly, these two higher-order mutants characterized previously were not sufficient to fully elucidate the roles of glycosylation on AGP biological function. To address such gene redundancy issues within the *GALTs*, we extended this work and characterized two additional higher-order *galt* mutants (*galt3 galt4 galt6* and *galt2 galt3 galt4 galt5 gal6*) generated by two CRISPR/Cas9 gene editing/multiplexing approaches along with the *galt2 galt5* T-DNA mutant. Our work has not only demonstrated the feasibility of applying CRISPR-Cas9 gene editing technology to create higher-order mutants for redundant gene families, but also offers insights into understanding the biological importance of sugar decorations on AGPs in plant growth and reproduction.

## Results

### Expression profiles of the five GALTs (GALT2 –GALT6)

Publicly available RNA-seq data generated from the Klepikova *Arabidopsis* Atlas eFP Browser (bar.utoronto.ca) was used to compare the expression profiles of the five *GALT* and three *HPGT* genes in different developmental stages in *Arabidopsis* [18]. Based on the RNA-seq data, *GALT2*, *GALT3*, *GALT5*, *HPGT1*, and *HPGT2* were expressed in roots, rosette leaves, inflorescences, and during seed germination and flower development. By contrast, *GALT4* and *GALT6* showed tissue-specific expression patterns. *GALT4* was expressed during flower development, whereas *GALT6* was only expressed in dry seeds, senescent leaf petioles, first internodes, and the first two siliques. Unlike *HPGT1* and *HPGT2* that are broadly expressed across tissues, *HPGT3* only had moderate expression in flowers and seeds. It is noteworthy that all the *GALT* and *HPGT* genes except for *GALT6* had relatively high expression values in sepals and carpels when compared to other tissues. As most *GALT* genes were expressed during flower development, they were expected to play a role in plant reproduction.

### Design of two CRISPR/Cas9 multiplexing constructs targeting *GALT3, GALT4,* and *GALT6* in *Arabidopsis*

**Fig. 1:**
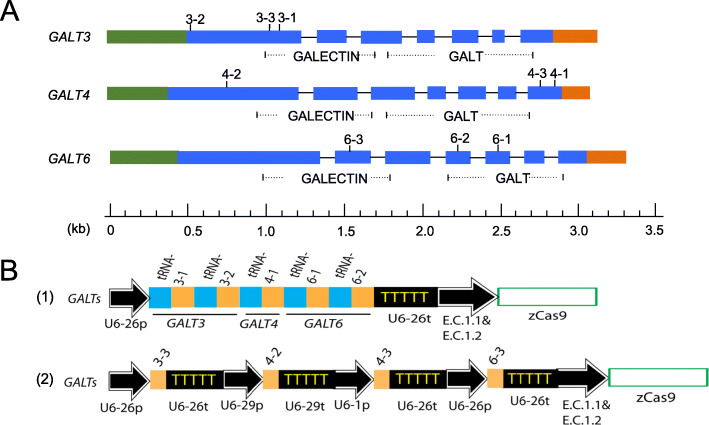
Schematic illustration of gRNA target sites and the two CRISPR multiplexing constructs. **a.** Three gRNAs were chosen for each of the *GALTs*, which belong to the five-membered galactosyltransferase (*GALT*) gene family. gRNAs were labeled as 3–1, 3–2, and 3–3 for *GALT3*; 4–1, 4–2, and 4–3 for *GALT4*; 6–1, 6–2, and 6–3 for *GALT6*; these gRNAs were used in gene constructs shown in 1**b**. (Green and orange represent 5′-UTR and 3′-UTR regions, respectively; blue represents exons; black lines represent introns. Online software named CRISPR-P 2.0 (http://crispr.hzau.cn/cgi-bin/CRISPR2/CRISPR) was used to design all gRNAs. Pfam domain predictions: Pf01762 corresponds to the Galactosyltransferase (GALT) domain; Pf00337 corresponds to the Galactose-binding lectin (GALECTIN) domain. **b-1.** Five gRNAs (3–1, 3–2, 4–1, 6–1, and 6–2 shown in 1**a**) with each gRNA fused with a tRNA were cloned in a single polycistronic transcription unit to target *GALT3*, *GALT4*, and *GALT6* simultaneously. **b-2.** Four gRNAs (3–3, 4–2, 4–3, and 6–3 in 1**a**) were assembled as four individual transcription units to target *GALT3*, *GALT4*, and *GALT6*. Both B-1 and B-2 were cloned in the pHEE401E vector, which contains a maize codon-optimized Cas9 gene (zCas9) driven by an Arabidopsis egg-cell specific promoter (E.C.1.1) fused with an egg-cell specific enhancer (E.C.1.2) [19]

**Fig. 2:**
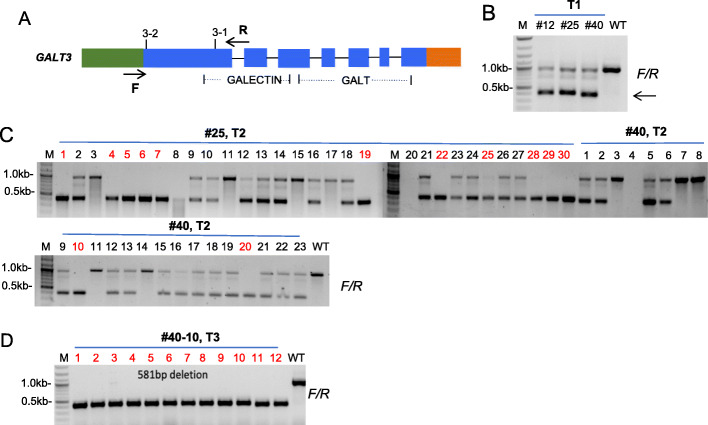
Inheritance patterns of CRISPR/Cas9 induced gene deletion of *GALT3* in the T1, T2, and T3 generations in selected transgenic lines. **a.** Locations of the two primers (F/R) used to amplify the gene fragment covering the two target sites (3–1 and 3–2) of *GALT3.* **b.** Three T1 CRISPR lines (#12, #25, and #40) containing partial deletions in *GALT3*. **c.** Segregation patterns of gene editing in two T2 transgenic lines (#25 and #40) showing partial and complete (red numbers) gene deletions of *GALT3*. **d.** Stable transmission of gene editing of the #40–10 transgenic line in the T3 generation showing complete deletions in all progeny. M corresponds to the DNA ladder, red numbers indicate transgenic lines that contained a full deletion in the *GALT3* gene

Previously, our lab characterized *galt2 galt5* T-DNA double mutants in *Arabidopsis*. To further reveal functional relevance between the *GALT* gene family and the glycosylation process of AGPs, we took advantage of the CRISPR/Cas9 multiplexing approach to further target additional *GALTs*. The *GALTs* are gene homologs from Clade 7 in the CAZy GT31 family and were previously demonstrated to have Hyp-*O*-GALT activity in *N. tabacum* [5, 6]. To target multiple *GALTs*, three guide RNA (gRNA) target sites per gene were chosen in *GALT3, GALT4,* and *GALT6* (Fig. 1a). These gRNAs were then assembled into two CRISPR constructs using two multiplexing methods. The method used for the first construct was based on a polycistronic tRNA-gRNA (PTG) system, which was originally developed for CRIPSR multiplexing in rice [20] (Fig. 1b-1). Once the PTG units are transcribed in plants, endogenous RNase Z and RNase P cleave at the tRNA site, releasing gRNAs for binding to their respective targets. Because the rice tRNA sequence used in the PTG construct is conserved in *Arabidopsis*, the PTG system has also been applied to *Arabidopsis* for multiplex gene editing [19]. The method used for the second construct involved four gRNA cassettes, each with its own promoter and terminator (Fig. 1b-2). The gRNAs in this second construct generally targeted more upstream regions of the three *GALT* genes. Both the first and second constructs were cloned in the pHEE401E vector, which contains a maize codon-optimized Cas9 gene (zCas9) driven by an *Arabidopsis* egg-cell specific promoter (E.C.1.1) fused with an egg-cell specific enhancer (E.C.1.2) [21].

**Fig. 3:**
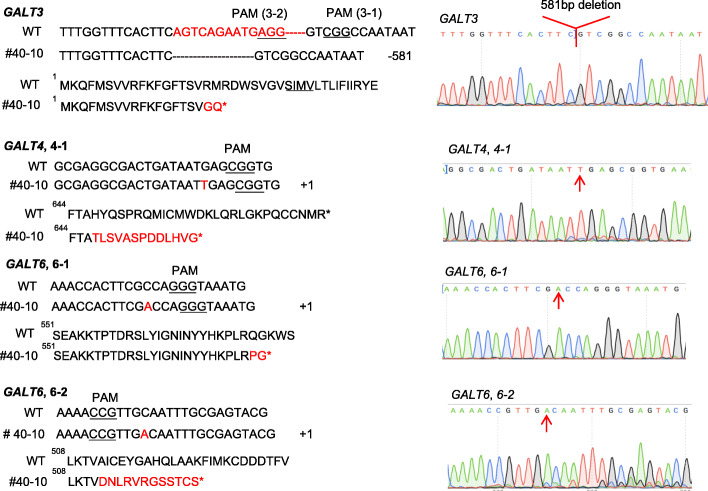
Efficient gene editing of all given target sites of *galt2 galt3 galt4 galt5 galt6* mutant line #40–10. (From top to bottom) Two gRNAs (3–1 and 3–2) created a 581 bp deletion in *GALT3*; *GALT4* contained a 1 bp insertion in the last exon; *GALT6* contained 1 bp insertions at both 6–1 and 6–2 target sites. All mutations resulted in frame-shifts and pre-mature stop codons. Mutation sites are indicated by red arrows on the sequencing chromatograms on the right

By transforming the tRNA-gRNA construct (Fig. 1b-1) into the *galt2 galt5* T-DNA double mutant background, 68 T1 transgenic lines were obtained. Of these 68 T1 lines, three of them (#12, #25, and #40) contained partial deletions created by 3–1 and 3–2 in *GALT3* (Fig. 2a and b). Surprisingly, none of the 68 T1 CRISPR lines screened showed a deletion between the 6–1 and 6–2 target sites in *GALT6*. In order to obtain mutants that contained a full deletion in *GALT3*, the two T1 CRISPR lines (#25 and #40) that contained partial mutations were selfed and used to produce T2 lines. In the T2, 80% of the progeny from #25 inherited the mutation from T1, including 36.7% of the progeny containing a 581 bp deletion in *GALT3* (Fig. 2c). Furthermore, 74% of the progeny from #40 inherited the mutation, including 8.7% of the progeny containing a 581 bp deletion in *GALT3* (Fig. 2c). Several lines that contained a 581 bp deletion in the T2 were selfed to produce the T3. In the T3, all of the 12 randomly chosen progenies from #40–10 inherited a 581 bp deletion in *GALT3* (Fig. 2d). It should be noted that #40–10 also contained homozygous gene-editing events in the *GALT4* and *GALT6* genes as well. *GALT4* contained a 1 bp insertion in its last exon created by 4–1 (Fig. 3). Unlike 3–1 and 3–2, the gene-editing events for 6–1 and 6–2 in #40–10 did not create a gene deletion between the two sites, but did result in 1 bp deletions at both sites in the *GALT6* gene (Fig. 3).

**Fig. 4:**
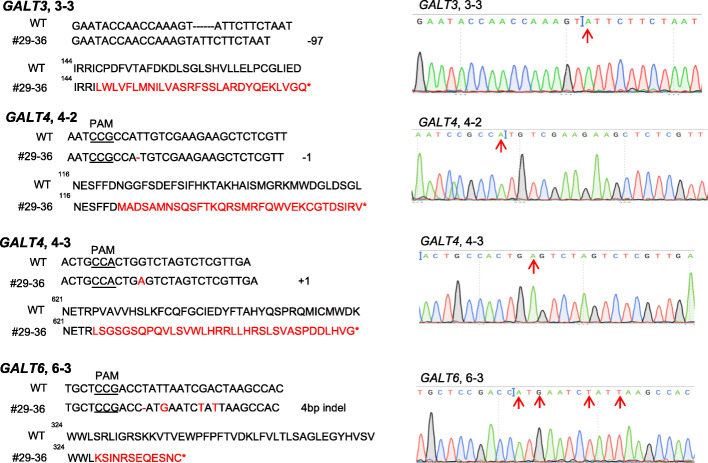
Multiplex gene-editing events occurred in three gRNAs of *galt2 galt3 galt4 galt5 galt6* mutant line #29–36. (From top to bottom) The 3–3 gRNA generated a 97 bp deletion in *GALT3*; the 4–2 and 4–3 gRNAs created a 1 bp deletion and a 1 bp insertion in the *GALT4* gene, respectively; the 6–3 gRNA produced a 4 bp indel mutation in the *GALT6* gene. All mutations resulted in frame-shifts (red amino acids) and pre-mature stop codons (asterisks) in their respective protein sequences. Mutation sites are indicated by red arrows on the sequencing chromatograms on the right

Because gRNAs for *GALT4* (4–1) and *GALT6* (6–1 and 6–2) in the tRNA-gRNA construct were located after the GALECTIN domain (Fig. 1a), we were not sure whether these target sites would fully eliminate GT activity from the corresponding *GALTs*. To ensure that mutants have both GALECTIN and GALT domains inactivated, another CRISPR construct was created that contained gRNAs (3–3, 4–2, 4–3, and 6–3 in Fig. 1a); these gRNAs all generally reside upstream of the two catalytic domains (GALECTIN and GALT) (Fig. 1b-2)*.* By transforming this gene construct into the *galt2 galt5* T-DNA homozygous mutant background, one quintuple mutant line #29–36 was obtained which contained homozygous mutations at all the gRNA target sites, included all three genes (*GALT3*, *GALT4*, and *GALT6*), and resulted in pre-mature stop codons (Fig. 4). Therefore, we used this #29–36 mutant line instead of the previous quintuple mutant line (#40–10) for biochemical and functional characterization.

### Quantification of β-D-Gal-Yariv precipitated AGPs in various *galt* mutants

**Fig. 5:**
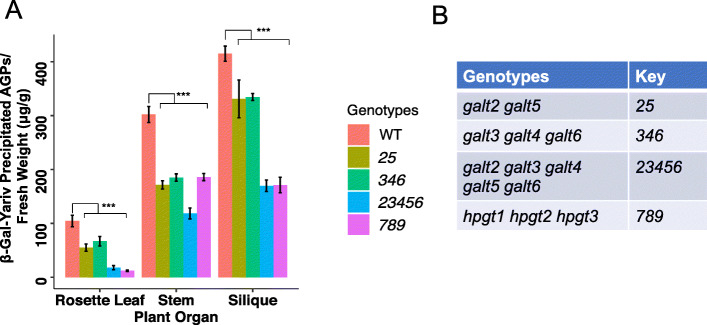
Quantification of AGPs in 40-day-old higher-order *galt* mutants in different organs by β-D-Gal-Yariv precipitation. **a** All higher-order mutants showed significant reductions in AGP content in rosette leaves, stems, and siliques. The *23456* and *789* mutants showed similar reductions in AGP content in rosette leaves and siliques. Both the *25 *and *346* mutants exhibited similar AGP content across tissues. Statistical differences were determined by two-way ANOVA, followed by the Tukey’s honestly significant difference test (***, *P* < 0.001). **b** Key to mutant genotypes shown in 5**a**

To examine whether the higher-order *galt* mutants contained less (glycosylated) AGPs, AGPs were extracted from rosette leaves, stems, and siliques from the *galt* mutants using β-D-Gal-Yariv reagent, which can specifically bind to the β-1,3-galactosyl sugar backbone of AGPs [22] and allow for their quantification [23]. For WT and the various *galt* mutants, AGPs were most abundant in siliques followed by stems and rosette leaves (Fig. 5). As expected, all the *galt* mutants exhibited significant reductions in AGPs. Both *23456* (quintuple mutant) and *hpgt1 hpgt2 hpgt3* T-DNA mutant (hereafter referred to as the *789* triple mutant) had similar reductions of AGPs in rosette leaves (80%) and siliques (55%) compared to that of WT, whereas *25* (i.e., the T-DNA double mutant) and the *346* triple mutant exhibited a lesser degree of AGP reduction in rosette leaves (40–45%), stems (30–40%), and siliques (15–16%) (Fig. 5)*.* The fact that *23456* showed considerably more AGP reductions than *25* and *346* reflects redundancy within the *GALT* gene family and emphasizes the value of the CRISPR-based approach to produce higher-order mutants to elucidate their biological functions.

### Monosaccharide composition analysis

**Fig. 6:**
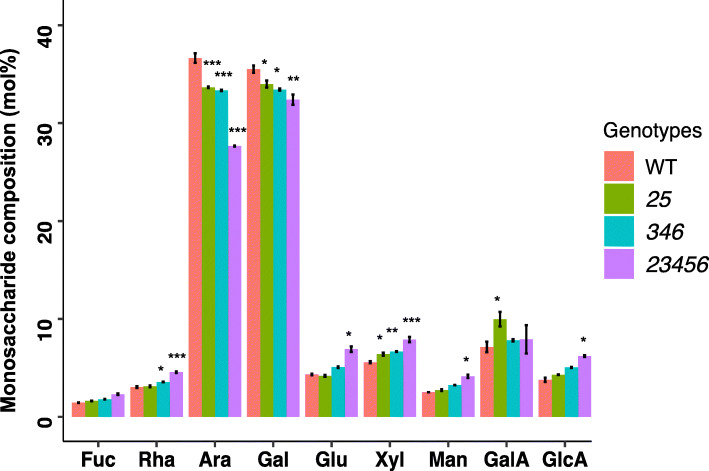
Monosaccharide composition analyses of AGPs extracted from five-week-old rosette leaves. Relative abundance of monosaccharide composition of AGPs extracted from *25* (*galt2 galt5*), *346 (galt3 galt4 galt6)*, *23456 (galt2 galt3 galt4 galt5 galt6)*, and WT calculated by mol%. Statistical differences were determined by two-way ANOVA, followed by the Tukey’s honestly significant difference test (*, *P* < 0.05; **, *P* < 0.01; ***, *P* < 0.001)

To examine the sugar composition of the AGPs in various *galt* mutants, we purified AGPs from five-week-old rosette leaves of the mutants using β-D-Gal-Yariv reagent followed by monosaccharide composition analysis by high performance anion exchange chromatography with pulsed amperometric detection (HPAEC-PAD). The *25* double mutant*, 346* triple mutant, and *23,456* quintuple mutant respectively showed ~ 8%, ~ 8%, and ~ 25% reductions in Ara compared to that of WT (Fig. 6)*.* Similarly, the Gal content was also respectively reduced by ~ 6%, ~ 8%, and ~ 10% in *25*, *346*, and *23,456* mutants compared to WT. The decrease in Ara and Gal consequently resulted in a relative increase of other monosaccharaides such as rhamose (Rha), xylose (Xyl), and glucuronic acid (GlcA) (Fig. 6).

### Root growth phenotypes of the *galt* mutants under normal conditions

**Fig. 7:**
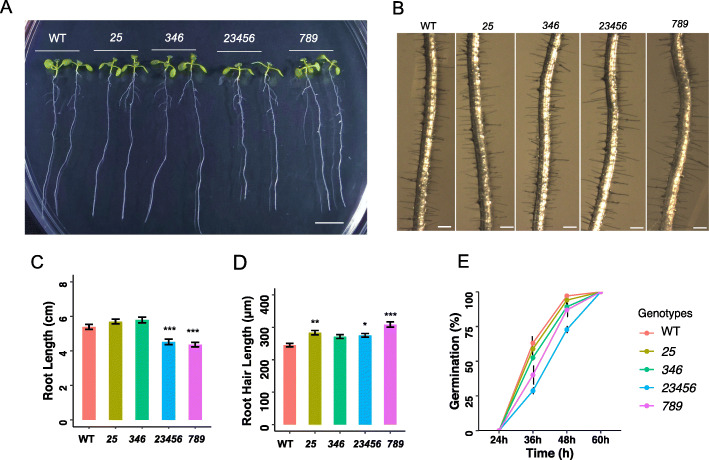
Root development and seed germination of various *galt* mutants. **a.** Photograph of nine-day-old *galt* mutants on 1/2 MS plates. Scale bar = 1 cm. **b.** Photograph of root hairs from nine-day-old *galt* mutants. Scale bar = 0.25 mm. **c.** Both *23456* (*galt2 galt3 galt4 galt5 galt6*) and *789 *(*hpgt1 hpgt2 hpgt3)* exhibited longer primary root lengths compared to WT. **d.** *25* (*galt2 galt5)*, *23456* (*galt2 galt3 galt4 galt5 galt6*) and *789* (*hpgt1 hpgt2 hpgt3)* exhibited longer root hairs compared to WT. **e.** Both *23456* (*galt2 galt3 galt4 galt5 galt6*) and *789* (*hpgt1 hpgt2 hpgt3)* showed a delayed germination phenotype. Statistical differences were determined by one-way ANOVA, followed by the Tukey’s honestly significant difference test (*, *P* < 0.05; **, *P* < 0.01; ***, *P* < 0.001)

To investigate whether knocking out multiple *GALT* genes affects *Arabidopsis* root development, primary root and root hair lengths were measured in the *galt* mutant and WT seedlings grown on ½ MS plates. Both the *23456* quintuple mutant and the *789* triple mutant exhibited shorter primary root length compared to WT, whereas both *25* and *346* showed similar root lengths as WT (Fig. 7a and c). Interestingly, root hairs of *25*, *23456*, and *789* were significantly longer compared to WT (Fig. 7b and d). In addition, both *23456* and *789* showed approximately a 12 h delay in seed germination compared to other *galt* mutants and WT, suggesting a key role for AGP glycosylation in seed germination (Fig. 7e).

### Root growth phenotypes of *galt* mutants in the presence of 30 μM β-D-Gal-Yariv

**Fig. 8:**
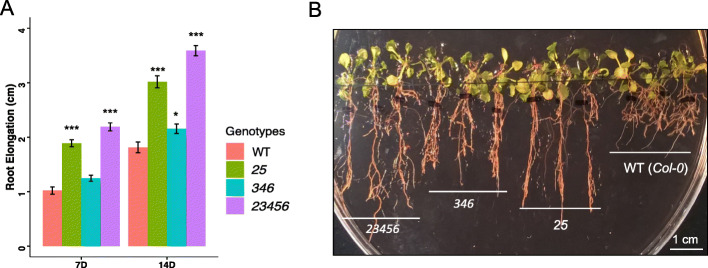
Root elongation of *galt* mutants in the presence of 30 μM β-D-Gal-Yariv. **a.** Root elongation of higher-order *galt* mutant and wild-type (*Col-0*) seedlings grown in the presence of 30 μM β-D-Gal-Yariv reagent for 7 and 14 days after transfer. All genotypes were germinated and grown on normal ½ MS for 4 days before being transferred onto ½ MS medium containing 30 μM β-D-Gal-Yariv reagent. Root lengths were measured 7 and 14 days after transfer (*, *P* < 0.05; ***, *P* < 0.001). **b.** Photograph of 14-day-old *galt* mutant and WT seedlings grown on ½ MS containing 30 μM β-D-Gal-Yariv reagent

Previous studies have shown that β-D-Gal-Yariv reagent can specially bind to β-1,3-galactose backbones on AGPs and inhibit cell division, root elongation, and pollen tube growth [23, 24]. To further verify the effect of β-D-Gal-Yariv on root growth of the *galt* mutants, four-day-old seedlings were transferred onto 1/2 MS plates containing 30 μM β-D-Gal-Yariv and grown for 2 weeks. Root elongation was measured 7 and 14 days after transfer. Both the *25* double mutant and the *23456* quintuple mutant showed much greater root elongation compared to WT and the *346* triple mutant after 7 and 14 days. The root length of the *346* mutant was significantly longer than WT after 14 days, but not after 7 days (Fig. 8).

### GALTs contribute to normal vegetative and reproductive growth in *Arabidopsis*

**Fig. 9:**
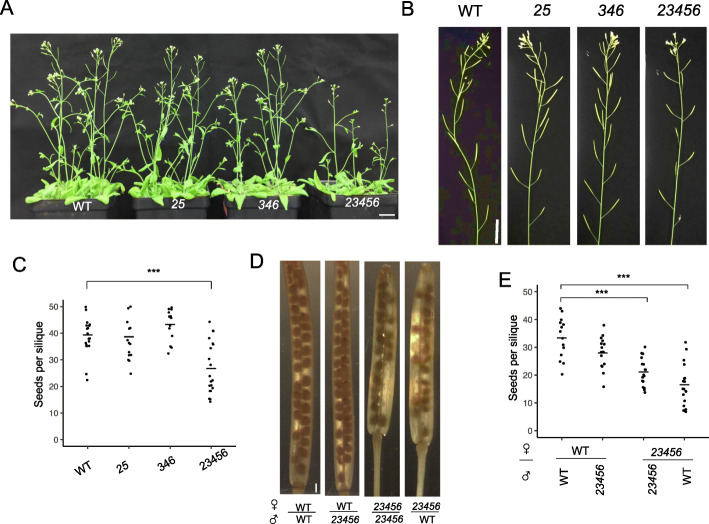
Overall plant growth and silique phenotypes displayed by the *galt* mutants. **a.** Shoots of five-week-old WT, *25* (*galt2 galt5*), *346* (*galt3 galt4 galt6*)*,* and *23456* (*galt2 galt3 galt4 galt5 galt6*) plants. Scale bar = 2 cm. **b.** Inflorescences of six-week-old WT, *25* (*galt2 galt5*), *346* (*galt3 galt4 galt6*)*,* and *23456* (*galt2 galt3 galt4 galt5 galt6*) plants. Scale bar = 2 cm. **c.** Quantification of seed set for WT, *25* (*galt2 galt5*), *346* (*galt3 galt4 galt6*)*,* and *23456* (*galt2 galt3 galt4 galt5 galt6*) plants (*n* = 15). **d.** Photograph of cleared siliques obtained by reciprocal crossing between WT and the *23456* (*galt2 galt3 galt4 galt5 galt6*) mutant. **e.** Quantification of seed set for reciprocal crossing between WT and the *23456* (*galt2 galt3 galt4 galt5 galt6*) mutant (*n* = 15) (***, *P* < 0.001)

**Fig. 10:**
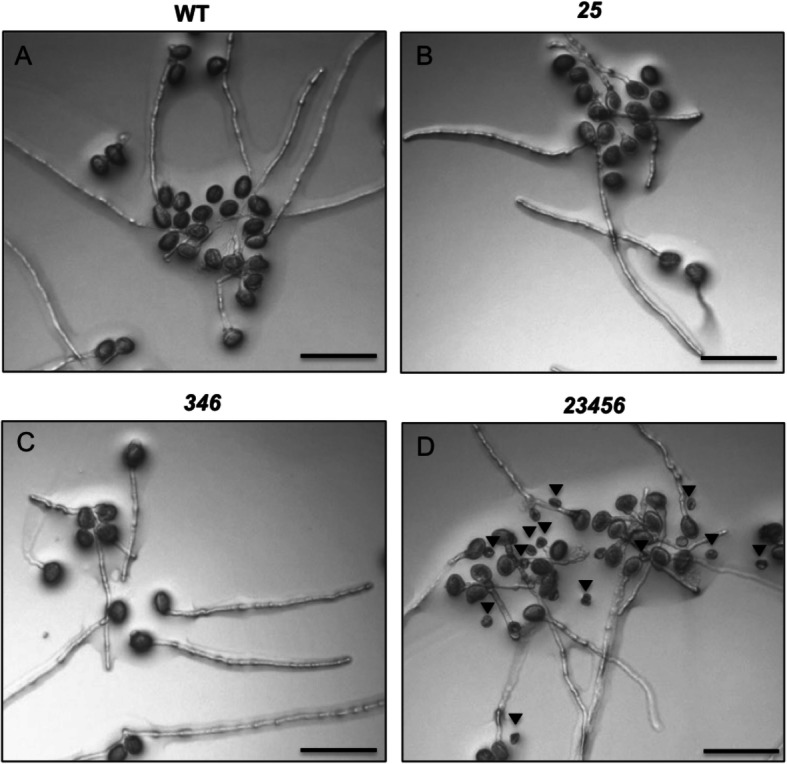
In vitro pollen germination of the *galt* mutants and WT. The *23456* mutant showed defective pollen as indicated by black arrowheads. All photographs were taken 3 h after incubation of pollen grains on pollen germination media. Scale bar = 100 μm

**Fig. 11:**
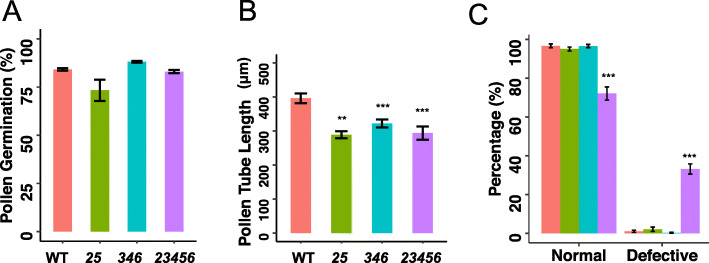
Pollen germination rate of normal pollen, pollen tube length, and defective pollen rate of higher-order *galt* mutants and WT 3 h after incubation of pollen grains on pollen germination media. **a**. All *galt* mutants showed similar pollen germination rates compared to WT. **b.** All the *galt* mutants showed shorter pollen tube lengths compared to WT. **c.** The *23456* quintuple mutant contained more defective pollen compared to the other *galt* mutants and WT (**, *P* < 0.01, ***, *P* < 0.001)

Phenotypically, the *25* double and *346* triple mutants were similar to WT except that these two mutants had 26% and 34% fewer siliques per plant (Fig. 9b and Supplemental Table 5). The *23456* quintuple mutant, however, displayed a number of abnormal growth phenotypes such as smaller and fewer rosette leaves, approximately a 2 day delay in flowering, a 16% reduction in plant height, 20% shorter siliques, 65% fewer siliques per plant, and a 30% reduction in seed set (Fig. 9 and Supplemental Table 5). In order to understand whether the male or female gametophyte of the *23456* mutant is responsible for reduced fertility, reciprocal crossing was done between *23456* and WT plants. When pollen from either WT or *23456* was used to pollinate WT plants*,* both silique length and seed set were normal. However, when pollen from either WT or *23456* was used to pollinate the *23456* mutant, shorter siliques and a reduced number of seeds were observed (Fig. 9d-e). These reciprocal crossing data indicated that shorter siliques and reduced seed set of *23456* are a result of defects in the female gametophyte. In vitro pollen germination was done to examine the pollen phenotypes of the various *galt* mutants. All the *galt* mutants contained normal shape pollen except for the *23456* mutant which had ~ 25% of its pollen being small, defective pollen that failed to germinate in vitro (Fig. 10 and 11c). Pollen germination rates for the normally shaped pollen in all the *galt* mutants were similar to that of WT (Fig. 11a). However, all the *galt* mutants exhibited an approximately 25% reduction in pollen tube length compared to WT (Fig. 11b).

## Discussion

### CRISPR-Cas9 gene editing facilitates study of GTs involved in AGP glycosylation

Arabinogalactan-proteins are the most highly glycosylated proteins in the plant kingdom [3]. Given that AGPs are composed of approximately 90–98% sugar, it is likely that the AG polysaccharide chains form the interactive molecular surface of these glycoproteins and control their biological functions [25]. Consequently, the GTs responsible for adding the sugars to AGPs are of particular interest. Studying these GTs can be particularly challenging for several reasons, including the finding that these GTs exist in gene families and have overlapping and redundant activities, which makes it difficult to examine their effects on AGP structure and function [26, 27].

**Fig. 12:**
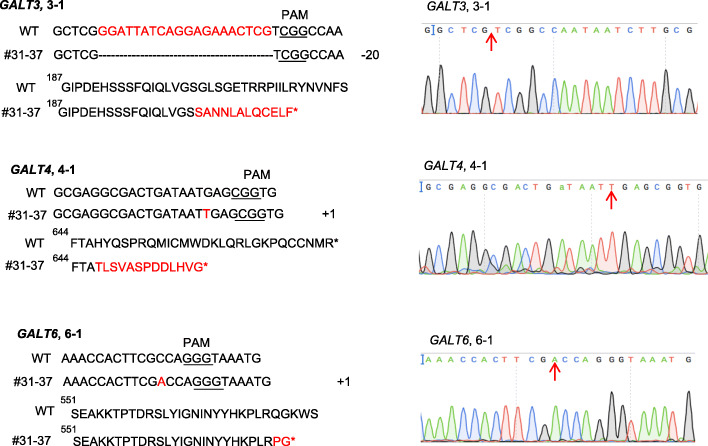
Gene editing in the *galt3 galt4 galt6* mutant line #31–37. Sequencing results of the *GALT3*, *GALT4*, and *GALT6* genes in the #31–37 line produced in the *Col-0* background. Mutations in *GALT3*, *GALT4*, and *GALT6* all resulted in pre-mature stop codons (see red amino acids and asterisks). Mutation sites are indicated by red arrows on the sequencing chromatograms on the right

Here, two multiplexing CRISPR constructs were generated to target multiple *GALT* genes (i.e., *GALT3*, *GALT4*, and *GALT6*) that are involved in Hyp-*O*-galactosylation of AGPs in *Arabidopsis*. Specifically, two homozygous mutant lines [i.e, a *346* triple mutant and a *23456* quintuple mutant] were selected for further characterization (Fig. 1b-1 and b-2). CRISPR-Cas9-induced mutations occurred in both mutants and resulted in premature stop codons for all the target sites (Figs. 12 and 4). Off-target gene mutations were identified by CRISPR-P 2.0 software. The five top off-target genes were identified and sequenced from the GALT CRISPR mutants. No off-target mutations were identified in the GALT CRISPR mutants (Supplemental Table 6).

The fewer phenotypic changes seen in the *25* double mutant and the *346* triple mutant compared to the *23456* quintuple mutant indicates that there is a compensation effect among the *GALTs*. Thus, it is only possible to reveal true biological functions of AGP sugar decorations by eliminating as many redundant genes as possible, preferably by using the CRISPR/Cas9 multiplexing approach. Given the five *GALTs* (*GALT2–6*) and three *HPGTs* (*HPGT1–3*) each belong to separate clades in GT31 and the *23456* CRISPR mutant displayed a pleiotropic phenotypes resembling the previously reported *789* T-DNA mutant, this indicates that each GT31 clade contributes significantly to the AGP sugar initiation step in *Arabidopsis* [7].

### CRISPR-induced mutations in the *GALT* genes result in less glycosylated AGPs and reductions in Ara and Gal

Significant reductions of glycosylated AGPs, as detected by β-D-Gal-Yariv precipitation, were found in the rosette leaves, stems, and siliques of all the *galt* mutants compared to WT (Fig. 5). Furthermore, monosaccharide analysis of the rosette leaf AGPs showed that the *23456* mutant showed the greatest reductions in arabinose (Ara) and galactose (Gal) compared to the *25* and *346* mutants, again illustrating the five *GALT* genes (*GALT2*, *GALT3*, *GALT4*, *GALT5*, and *GALT6*) are functionally redundant (Fig. 6). These data are consistent with the idea that the initiation of galactosylation of Hyp residues in AGP protein cores is increasingly reduced as more and more *GALT* genes are knocked out, resulting in AGP molecules that are less glycosylated overall because they have fewer Hyp-AG chains on their polypeptide backbones. The AG chains that are added in these mutants are likely similar to wild type AG chains, but do show some reduced amounts of Gal and Ara, which may reflect reduced activities of other potentially interacting GALTs (i.e., β-1,3-galactosyltransferases involved in chain elongation and β-1,6-galactosyltransferases involved in side chain formation/elongation) and arabinosyltransferases when the *Hyp-GALT* genes are knocked-out. Future research can focus on such potential protein-protein interactions among the GTs responsible for AGP biosynthesis.

### AGP glycosylation contributes to normal root growth in *Arabidopsis*

Both the *23456* CRISPR quintuple mutant and the *789* T-DNA triple mutant exhibited shorter root lengths as well as longer root hairs when grown under normal conditions, indicating some degree of gene redundancy between the *GALTs* and *HPGTs* (Figs. 7 and 8). When treated with 30 μM β-D-Gal-Yariv reagent, the *23456* mutant exhibited the greatest root elongation followed by *25*, *346*, and WT (Fig. 9). The significantly longer roots shown by *25* compared to *346* after Yariv treatment indicates that *GALT2* and *GALT5* may play more substantial roles in the glycosylation of AGPs in the root compared to *GALT3*, *GALT4*, and *GALT6* (Fig. 9). This also indicates a partial gene redundancy exists among the *GALTs* depending on the organ type. This is in agreement with previous findings that single *galt* mutants such as *galt2*, *galt3*, *galt4*, *galt5*, and *galt6* were shown to be less sensitive to Yariv inhibition of root growth [6]. In addition, the *789* mutant was previously reported to have longer lateral roots and increased root hair lengths compared to WT [7]. As suggested by Basu et al. [4], plants need to maintain a threshold level of glycosylated AGPs for normal growth, especially for roots which are particularly sensitive to AGP changes. Thus, reduced AGP glycosylation can impair root growth as shown in the *23456* and *789* mutants.

AGPs are well known to be localized and involved in root growth from a variety of studies. Yariv treated *Arabidopsis* roots exhibit a short, swollen root phenotype, which is caused by an inhibition of cell proliferation and cell elongation [23]. Several other AGP-related GTs or AGP mutants such as *reb1–1*, *ray1*, *fut4/fut6*, *glcat14a-1*, *fla1*, *fla4/sos5* and *agp30*, also display abnormal root phenotypes under normal or salt-stress conditions in *Arabidopsis* [12–14, 26–29]. In addition, AGPs antibodies such as JIM8, JIM13, and LM2 localize AGPs in primary roots, root caps, and root hairs [30–32]. The CRISPR mutants examined here clearly elucidate the important role of AGP glycosylation in root growth.

### AGP glycosylation is critical for normal growth and development

The *23456* CRISPR mutant displayed more abnormal above ground growth phenotypes when compared to the *25* mutant, the *346* mutant, and wild type plants (Fig. 9a-b; Supplemental Table 5). This abnormal growth phenotype included smaller and fewer rosette leaves as well as shorter inflorescences and was reminiscent of the dwarf phenotypes reported previously for the *789* triple mutant and the *ray1* mutant [7, 14]. The mechanism by which AGP glycosylation affects and controls such plant growth is largely unknown. One possibility, however, is that AG chains on AGPs initiated by the GALTs allow AGPs to be associated with other plasma membrane and cell wall components to maintain cell wall integrity. Many AGPs are located on the outer surface of the plasma membrane via GPI anchors, these AGPs are in close proximity with other plasma membrane proteins such as wall-associated kinases (WAKs), RLKs (e.g., FEI1/FEI2), and cellulose synthases [10, 28]. Such plasma membrane AGPs are also associated with the cytoskeleton, but the exact molecular interactions are unknown. By expressing a tomato AGP fusion protein (i.e., LeAGP1-GFP) in tobacco BY2 cells, LeAGP1 was found localized at the Hechtian strands during plasmolysis [31].

AGPs are also present in the cell wall and can interact with other wall components. Evidence of the binding between AGPs and pectin was suggested when AGPs were detected from a crude pectin fraction using AGP antibodies [32]. Addition of calcium can promote such binding between carrot AGPs and pectin, whereas EGTA (a calcium chelator) weakened the binding, indicating that calcium may serve as an important cross-linking agent [32]. It was proposed that AGPs and pectin serve as cell wall plasticizers that facilitate the binding among cell wall polysaccharides [33]. Perhaps the most dramatic example of AGPs interacting with other wall molecules is exhibited by the finding that a particular AGP known as APAP1 covalently interacts not only with pectin, but also with arabinoxylan [34]. Linkage analysis of APAP1 purified from *Arabidopsis* cell suspension cultures found covalent linkages between the AGP sugars and the sugars found in arabinoxylan and pectin (RG-I) [34]. Therefore, knocking out the *GALT* genes could affect these various associations between the carbohydrate residues on AGPs and their interacting molecules so as to alter cell wall network connections, leading to abnormal growth.

### AGP glycosylation functions in both male and female gametophyte development

Reciprocal crossing experiments showed that defects in the female gametophyte are responsible for the reduced seed set (~ 30% less) exhibited by the *23456* quintuple mutant (Fig. 9c-e). The more severe reduction in seed set in the *23456* mutant compared to *galt4* and *galt6* single mutants suggested *GALT4* and *GALT6* play redundant roles in seed development. Unlike these *galt* mutants*,* the *789* triple mutant was reported to have only shorter siliques, but normal seed set [7]. Thus, despite being Hyp-*O*-GALTs, the GALTs (GALT2–6) and the HPGTs (HPGT1–3) each may have partially overlapping roles in AGP glycosylation and function. The functional roles of GALTs in silique development demonstrate that the extent to which AGP glycosylation in the ovary contributes to normal female gametophyte development in *Arabidopsis*. As AGPs and their carbohydrate moieties are abundant in reproductive tissues such as pollen, pollen tubes, and ovaries, our results illustrate that the sugar moieties on AGPs initiated by the GALTs are essential for AGP functions relating to reproductive processes.

Several AGPs are known to be involved in female gametophyte development in *Arabidopsis*. AGP4 (JAGGER), was recently shown to act in synergid cells to block and prevent polytubey in *Arabidopsis* [35]. In the *jagger* mutant, the second synergid cell was not degenerated after fertilization and can continue to attract pollen tubes towards the embryo sac [35]. It was suggested that either the AGP4/JAGGER protein itself or the carbohydrate on AGP4/JAGGER serves as a signaling molecule that leads to the degeneration of the second synergid cell after fertilization and blocks more pollen from entering [35]. AGP19 is another AGP which is expressed in the ovary, transmission tract, and siliques of *Arabidopsis*. An *agp19* knockout mutant showed fewer and shorter siliques, indicating AGP19 functions in reproduction [36].

The carbohydrate portion of AGPs have been viewed as signaling molecules and nutrition sources during reproductive development including pollination, male and female gametophyte development, ovary guidance, fertilization, and embryogenesis [35–37]. One study found that the glycosylation of AGP18 established the transition from sporophytic to gametophytic tissue. Although the transcript of AGP18 is found in the egg cell, glycosylated AGP18 was only found in the functional megaspore mother cell [28]. A classic example of AGP glycosylation being involved in pollen-ovary communication is the Transmitting Tissue-Specific (TTS) protein, which is an AGP expressed in the transmitting tract of tobacco styles [38]. It was observed that pollen tubes were attracted and grew towards the higher concentrations of glycosylated TTS protein. Both in vitro and in vivo experiments found that pollen tubes can de-glycosylate TTS as it grows towards the ovary, suggesting that the carbohydrate on TTS signaled the way for pollen tubes to grow towards the ovary [38]. The finding that the polysaccharides on AGPs served as a pollen tube attractant in *Arabidopsis* is also consistent with another study done in *Torenia fournieri*. It was found that β-methyl-glucuronosyl galactose (4-Me-GlcA-β-1,6-Gal) from AGP glycan chains secreted from the ovaries of *Torenia fournieri* was essential for making pollen tubes competent for ovule targeting/guidance [39].

With respect to pollen development, most *galt* mutants except for *23456* contained normally shaped pollen (Fig. 11). All the *galt* mutants exhibited shorter pollen tubes compared to WT. In addition, approximately 30% of the pollen in the *23456* quintuple mutant was defective such that they were much smaller and non-viable (Fig. 11a-c). This defective pollen phenotype shown by the *23456* mutant phenocopied *Arabidopsis* RNAi mutants of *FLA3*, which encodes a fasciclin-like AGP, where half of the pollen grains were shrunken and non-viable [40]. The defective pollen in *fla3* showed abnormal pollen intine formation that led to defects in male fertility and reduced seed set [40]. *Arabidopsis* mutants such as *agp6, agp11*, and *agp6 agp11* also exhibited collapsed pollen, less pollen germination, reduced pollen tube elongation, and pollen release [40–42]. Another member of the GT31 family known as KNS4, was recently identified to have AGP β-(1,3)-galactosyltransferase activity and be  involved in the formation of the exine layer of *Arabidopsis* pollen [43]. The *kns4* T-DNA insertion mutant exhibited collapsed pollen grains which contained a thin exine wall and failed to separate from the pollen mother cells at the tetrad stage of pollen development [9]. It is likely that the defective pollen phenotype of the *23456 *is likewise caused by abnormal microspore wall formation. Similar phenotypes exhibited by individual AGP mutants and the *galt* mutants could be attributed to improperly glycosylated or under glycosylated AGPs. A previous study with the *galt* single mutants showed no difference in pollen shape and pollen tube elongation in *galt2*, *galt3*, *galt4*, *galt5*, and *galt6* mutants [6]. These contrasting findings are again indicative of gene redundancy within the *GALT* gene family.

Other possible roles for AGPs and/or the glycan chains on AGPs were investigated by immunolocalization in numerous plant species [44] and could be an area of future research. The development of AGP monoclonal antibodies (e.g. JIM8, JIM13, MAC207, and LM2) is a milestone in AGP research as it provides information on the localization and putative roles of AGPs and/or the complicated glycan structures on AGPs in specific cell types or developmental stages. Antibodies such as JIM8 and JIM13 have detected AGPs in the cell walls of microspores in *Arabidopsis* [45]. LM2 and MAC207 antibodies localized AGPs at the tips of pollen tubes in *Arabidopsis* [46]. By using JIM8 and JIM13 antibodies along with β-Glc-Yariv reagent, AGPs were found to be on the stigma only before pollination but were depleted after pollination in apple and Magnolia [46–48]. Similarly, JIM13-labeled AGPs showed AGPs signaled the way for growing pollen tubes in the micropylar canal; however, the AGP signal was depleted after pollen tube passage in apple. JIM8-labeled AGPs accumulated before and during pollination of apple ovaries, but soon vanished after fertilization [49].

## Conclusions

This research has successfully utilized the CRISPR-Cas9 multiplex gene editing technology for the generation of higher-order mutants [i.e. one triple (*galt3 galt4 galt6*) mutant and one quintuple (*galt2 galt3 galt4 galt5 galt6*) mutant] in the *GALT* gene family. Both biochemical and physiological analyses indicated gene redundancy exists within the *GALT* gene family. Biochemical analyses demonstrated the number of GALTs have an additive effect on the extent of glycosylation on AGPs. Phenotypic analyses revealed that AGP glycosylation is crucial for normal vegetative and reproductive growth, especially in root and shoot growth, pollen and pollen tube formation, female gametophyte development, seed set, and silique development in *Arabidopsis*. Future work can focus on dissecting the molecular mechanism(s) by which AGP glycosylation controls these various physiological processes. Although both the GALTs (GALT2–5) and the HPGTs (HPGT1–3) are involved in initiating glycosylation of AGPs, the GALTs contain both a GALECTIN domain and a GALT domain, while the HPGTs only contain a GALT domain. Future research should also focus on understanding the role of these two protein domains (i.e., GALECTIN and GALT) and addressing the evolutionary relationship between the GALTs and HPGTs. To gain further insight into elucidating the functional importance of glycosylation on AGPs, future work can also focus on the generation of additional higher-order mutants, where different or more genes in the eight membered *Hyp-O-GALT* gene family are knocked-out using our CRISPR-Cas9 multiplexing approach.

## Methods

### Plant material

*A. thaliana* (Columbia-0 ecotype) was obtained from the Arabidopsis Biological Research Center (ABRC), Columbus, Ohio, USA and used as WT. The *galt2 galt5* homozygous T-DNA mutant was previously generated in our lab [6]. The *hpgt1 hpgt2 hpgt3* homozygous T-DNA mutant was obtained from Dr. Ogawa-Ohnishi’s lab [7]. We generated all the CRISPR mutants in this study using this *Col-0* background.

### Vector constructions

An online gRNA designing tool CRISPR-P 2.0 (http://crispr.hzau.edu.cn/CRISPR2/) was used for gRNA design [49]. Five gRNAs were designed to target *GALT3*, *GALT4*, and *GALT6* simultaneously using the polycistronic tRNA-gRNA (PTG) method (Table S1) [20]. Each gRNA was amplified as a polycistronic tRNA-gRNA (PTG) unit from a template plasmid pGTR using primers listed in Tables S2 and S3. The pGTR plasmid was a gift from Dr. Yinong Yang’s lab (Addgene plasmid # 63143). After amplification, PCR products were purified by DNA gel extraction using the Wizard® SV gel and PCR clean-up kit from Promega. Six PTG units were ligated together during BsaI digestion, and a PCR reaction was performed to amplify the ligated PTG units using a universal primer pair (pHEE_S5AD-F and pHEE_S5AD-R). The ligated PTG PCR product was purified by DNA gel extraction using the Wizard® SV gel and PCR clean-up kit from Promega, and subsequently ligated to the binary vector pHEE401E, which was a gift from Dr. Qi-Jun Chen’s lab (Addgene plasmid #71287). The pHEE401E binary vector had a maize codon optimized *Cas9* gene driven by egg cell-specific promoters (E.C 1.1 and E. C 1.2) [50].

In the second construct designed to target *GALT3*, *GALT4*, and *GALT6*, four gRNAs were designed (Table S1). Primers used to amplify these gRNAs were listed in Table S4. After PCR amplifications, these gRNAs fragments were subsequently cloned into the BsaI site of the pHEE401E binary vector by the Golden Gate cloning method [51]. Three template plasmids (pCBC-DT1, pCBC-DT2T3, and pCBC-DT3T4) were gifts from Dr. Qi-Jun Chen’s lab (Addgene plasmid #50590; #50591; #50592) [21].

After both constructs were cloned into the pHEE401E vector and confirmed by Sanger sequencing, they were transformed into Agrobacterium strain GV3101, which was then used to transform the *Arabidopsis Col-0* ecotype via the floral dip method [52]. A simple PCR-based method was first used for detecting indel mutations and then confirmed by Sanger sequencing [53].

### AGPs quantification by β-D-Gal-Yariv

AGPs were extracted from four-week-old *galt* mutants and WT using β-D-Gal-Yariv [54]. Briefly, approximately 0.3 g of rosette leaves, stems, and siliques of the mutant and WT plants were pulverized using liquid nitrogen before addition of 1 mL 2% CaCl_2_ by gentle shaking for 2 h. Next, the supernatant was separated from the plant debris by centrifugation at 13,000 *g* for 10 min. Then, 200 μL β-D-Gal-Yariv reagent was added to the supernatant in a new 1.5 mL centrifuge tube; AGPs were allowed to precipitate for 2 h. To quantify AGP content, the precipitated AGP pellet was dissolved in 20 mM NaOH and measured using a UV-spectrometer at OD_420_. An AGP standard curve was made using gum arabic under the same Yariv precipitation conditions.

### Monosaccharide composition analysis by high pH anion exchange chromatography with pulsed amperometric detection (HPAEC-PAD)

Five-week-old rosette leaves from the *galt* mutants and WT were used for AGPs extraction. Plant tissues were first pulverized by liquid nitrogen and then mixed with 2% NaCl by gentle shaking for 2–3 h. Next, the supernatant was obtained by centrifugation at 13,000 *g* for 30 min. After that, β-D-Gal-Yariv reagent was added to the supernatant to allow for AGP precipitation overnight. The precipitated AGP pellet was obtained by low-speed centrifugation (2000 *g* − 5000 *g*) for 10 min and resuspended with distilled H_2_O. To break the linkage between Yariv and the AGPs, approximately 25 mg sodium dithionite was added to the AGP suspension and incubated at 50 °C for 10–15 min. Each AGP sample was passed through a PD-10 desalting column (GE Healthcare). The desalted AGPs were then freeze-dried. Monosaccharide composition analysis was performed using the same method described previously [17, 55].

### Measurements of root elongation and root hair length

For root growth measurement, seeds of the *galt* mutants and WT were first germinated on ½ MS agar plates. Young seedlings were then transferred onto new ½ MS agar plates on the fourth day after sowing and were allowed to grow vertically in a growth chamber at 22 °C, 16 h light/20 °C, 8 h dark photoperiod. Both root length and root hair length were measured 4 days after transfer. Root hairs were measured 5 mm from the root tip. Approximately 300 root hairs were measured using a Nikon SMZ1500 stereomicroscope at 20x magnification. For β-D-Gal-Yariv treatment, five-day-old seedlings were transferred onto ½ MS agar plates supplemented with 30 μM β-D-Gal-Yariv. Root elongation was measured 7 days and 14 days after transfer. Approximately 20 plants for each genotype were used for each measurement with three replicates.

### Germination experiment

Freshly harvested seeds of *galt* mutants and wild-type plants were first stratified at 4 °C for 3 d before being sown onto ½ MS and 1% sucrose agar plates. Germination percentages were counted at 24 h, 36 h, 48 h, and 60 h after sowing. Approximately 60 seeds were sown for each genotype with four replicates.

### Seed set measurements and reciprocal cross analysis

Mature siliques were harvested from five-week-old *galt* mutants and WT plants. Ten plants for each genotype were used for seed set measurements. The reciprocal crosses were done using stage 12 flowers for WT and the *galt2 galt3 galt4 galt5 galt6* mutants. For each reciprocal pair, approximately 15–20 successfully hand-pollinated siliques were chosen for seed set measurements. To measure seed numbers, the siliques were cleared with 70% ethanol for 2–3 days before being transferred into 50% glycerol. After clearing, seed numbers were counted using a Nikon SMZ1500 stereomicroscope at 10x magnification.

### In vitro pollen germination assay

In vitro pollen germination was performed using flowers from five-week-old *galt* mutants and WT. The method for in vitro pollen germination was described previously [17]. After incubating pollen on pollen germination medium for 3 h, pollen shape, the pollen germination rate, and pollen tube length were measured using a Nikon phot-lab2 microscope at 50x magnification. Approximately 300 samples were measured for each genotype with three replicates.

## Supplementary Information

**Additional file 1: Supplemental Table 1.** List of guide RNA sequences and their target genes.

**Additional file 2: Supplemental Table 2.** Names of the primer pairs for amplifying each polycistronic tRNA-guide RNA (PTG) unit.

**Additional file 3: Supplemental Table 3.** List of primer sequences for PTG cloning.

**Additional file 4: Supplemental Table 4.** List of primer sequences used to clone the second CRISPR multiplexing construct.

**Additional file 5: Supplemental Table 5.** Quantitative data of phenotypes of WT, *25*, *346*, and *23456* mutants.

**Additional file 6: Supplemental Table 6.** List of primers for sequencing off-targets of the *GALT* genes.

**Additional file 7: Supplemental Figure 1.** Gene editing events of *GALT3* generated by gRNA 3-1 and gRNA 3-2 were inherited to the next generation. (A-B) Segregation patterns of gene editing events of *GALT3* in two transgenic lines (#25 and #40 ) in the T2 generation; (C) (Left panel) Gene deletion of *GALT3* was inherited to the T3 generation in the #40-10 CRISPR line; (Right panel) Gene editing events of *GALT3* were detected in three independent transgenic lines (#12, #25 and #40) in the T1 generation. Red indicated a full deletion in the *GALT3* gene. **Supplemental Figure 2.** Growth phenotypes of the *galt* mutants in soil. Photographs of the *galt* mutants and WT were taken from the third week to the fifth week after sowing. Scale bar = 2 cm.

## Data Availability

The datasets used and/or analyzed during the current study are available from the corresponding author upon reasonable request.

## Abbreviations

HRGP: Hydroxyproline-rich glycoprotein
AGP: Arabinogalactan-protein
Hyp-*O*-GALT: Hydroxyproline-*O*-galactosyltransferase
Gal: Galactose
CAZy: Carbohydrate Active Enzyme (database)
GT: Glycosyltransferase
Guide RNA: gRNA
Hyp: Hydroxyproline
