# Supplementary material for: CRISPR-Cas9 multiplex genome editing of the hydroxyproline-O-galactosyltransferase gene family alters arabinogalactan-protein glycosylation and function in Arabidopsis
Source: BMC Plant Biol. 2021 Jan 6;21:16. doi: 10.1186/s12870-020-02791-9 (PMC7789275; doi:10.1186/s12870-020-02791-9)
Supplement: Supplementary file 1 — Additional file 1: Supplemental Table 1. List of guide RNA sequences and their target genes. [file 12870_2020_2791_MOESM1_ESM.pdf]

Supplemental Table 1. List of guide RNA sequences and their target genes.

**Target site (Target gene)**	**gRNA sequence**
3-1 (*GALT3*)	GATTATCAGGAGAAACTCGT**CGG**
3-2 (*GALT3*)	TTTCACTTCAGTCAGAATGA**GGG**
3-3 (*GALT3*)	GTACTTCTTGAGCTTCCTTG**TGG**
4-1 (*GALT4*)	GCGAGGCGACTGATAATGAG**CGG**
4-2 (*GALT4*)	GAGAGCTTCTTCGACAATGG**CGG**
4-3 (*GALT4*)	GTTCAACGAGACTAGACCAG**TGG**
6-1 (GALT6)	TATCACAAACCACTTCGCCA**GGG**
6-2 (GALT6)	CCCGTACTCGCAAATTGCAA**CGG**
6-3 (GALT6)	GGCTTAGTCGATTAATAGGT**CGG**
