# Supplementary material for: CRISPR-Cas9 multiplex genome editing of the hydroxyproline-O-galactosyltransferase gene family alters arabinogalactan-protein glycosylation and function in Arabidopsis
Source: BMC Plant Biol. 2021 Jan 6;21:16. doi: 10.1186/s12870-020-02791-9 (PMC7789275; doi:10.1186/s12870-020-02791-9)
Supplement: Supplementary file 2 — Additional file 2: Supplemental Table 2. Names of the primer pairs for amplifying each polycistronic tRNA-guide RNA (PTG) unit. [file 12870_2020_2791_MOESM2_ESM.pdf]

**Supplemental Table 2.** Names of the primer pairs for amplifying each polycistronic tRNA-guide RNA (PTG) unit.

| **Forward Primer** | **Reverse Primer** | **PTG units** |
|---|---|---|
| pHEE_L5AD5-F | GALT3-2_gR1_R | PTG 1 |
| GALT3-2_gR1_F | GALT3-1_gR2_R | PTG 1-2 |
| GALT3-1_gR2_F | GALT4-1_gR3_R | PTG 2-3 |
| GALT4-1_gR3_F | GALT6-2_gR4_R | PTG 3-4 |
| GALT6-2_gR4_F | GALT6-1_gR5_R | PTG 4-5 |
| GALT6-1_gR5_F | L5AD5-R | PTG 5 |
