# Supplementary material for: CRISPR-Cas9 multiplex genome editing of the hydroxyproline-O-galactosyltransferase gene family alters arabinogalactan-protein glycosylation and function in Arabidopsis
Source: BMC Plant Biol. 2021 Jan 6;21:16. doi: 10.1186/s12870-020-02791-9 (PMC7789275; doi:10.1186/s12870-020-02791-9)
Supplement: Supplementary file 3 — Additional file 3: Supplemental Table 3. List of primer sequences for PTG cloning. [file 12870_2020_2791_MOESM3_ESM.pdf]

Supplemental Table 3. List of primer sequences for PTG cloning.

Primer Name	Sequence
pHEE_L5AD5-F	CGGGTCTCGATTGGGATGGGCAGTCTGATTGACAAAGCACCAGTGG
GALT3-2_gR1_R	ATGGTCTCAACTGAAGTGAAATGCACCAGCCGGGAA
GALT3-2_gR1_F	TAGGTCTCCCAGTCAGAATGAGTTTTAGAGCTAGAA
GALT3-1_gR2_R	ATGGTCTCACTCCTGATAATCTGCACCAGCCGGGAA
GALT3-1_gR2_F	TAGGTCTCCGGAGAAACTCGTGTTTTAGAGCTAGAA
GALT4-1_gR3_R	ATGGTCTCACAGTCGCCTCGCTGCACCAGCCGGGAA
GALT4-1_gR3_F	TAGGTCTCCACTGATAATGAGGTTTTAGAGCTAGAA
GALT6-2_gR4_R	ATGGTCTCATGCGAGTACGGGTGCACCAGCCGGGAA
GALT6-2_gR4_F	TAGGTCTCCCGCAAATTGCAAGTTTTAGAGCTAGAA
GALT6-1_gR5_R	ATGGTCTCATGGTTTGTGATATGCACCAGCCGGGAA
GALT6-1_gR5_F	TAGGTCTCCACCACTTCGCCAGTTTTAGAGCTAGAA
pHEE_L5AD5-R	TAGGTCTCCAAACGGATGAGCGACAGCAAACAAAAAAAAAAGCACC GACTCG
pHEE_S5AD5-F	CGGGTCTCAATTGGGATGGGCAGTCTGATTG
pHEE_S5AD5-R	TAGGTCTCCAAACGGATGAGCGACAGCAAAC
