# Supplementary material for: CRISPR-Cas9 multiplex genome editing of the hydroxyproline-O-galactosyltransferase gene family alters arabinogalactan-protein glycosylation and function in Arabidopsis
Source: BMC Plant Biol. 2021 Jan 6;21:16. doi: 10.1186/s12870-020-02791-9 (PMC7789275; doi:10.1186/s12870-020-02791-9)
Supplement: Supplementary file 4 — Additional file 4: Supplemental Table 4. List of primer sequences used to clone the second CRISPR multiplexing construct. [file 12870_2020_2791_MOESM4_ESM.pdf]

Supplemental Table 4. List of primer sequences used to clone the second CRISPR multiplexing construct.

Primer Name	Sequence
DT1-BsF_3-3	ATATATGGTCTCGATTGTACTTCTTGAGCTTCCTTGGTT
DT1-F0_3-3	TGTACTTCTTGAGCTTCCTTGGTTTTAGAGCTAGAAATAGC
DT0-BsR2	ATATTATTGGTCTCAATCTCTTAGTCGACTCTACCAAT
DT2-BsF2_4-2	ATATTATTGGTCTCAAGATTGAGAGCTTCTTCGACAATGGGTT
DT2-F0_4-2	TGAGAGCTTCTTCGACAATGGGTTTTAGAGCTAGAAATAGC
DT0-BsR3	ATATTATTGGTCTCATCACTACTTCGTCTCTAACCAT
DT3-BsF3_4-3	ATATTATTGGTCTCAGTGATTGTTCAACGAGACTAGACCAGGTT
DT3-F0_4-3	TGTTCAACGAGACTAGACCAGGTTTTAGAGCTAGAAATAGC
DT4-R0_6-3	AACACCTATTAATCGACTAAGCCAATCACTACTTCGACTCTAGCTGTAT
DT4-BsR_6-3	ATTATTGGTCTCTAAACACCTATTAATCGACTAAGC
