# Supplementary material for: CRISPR-Cas9 multiplex genome editing of the hydroxyproline-O-galactosyltransferase gene family alters arabinogalactan-protein glycosylation and function in Arabidopsis
Source: BMC Plant Biol. 2021 Jan 6;21:16. doi: 10.1186/s12870-020-02791-9 (PMC7789275; doi:10.1186/s12870-020-02791-9)
Supplement: Supplementary file 5 — Additional file 5: Supplemental Table 5. Quantitative data of phenotypes of WT, 25, 346, and 23456 mutants. [file 12870_2020_2791_MOESM5_ESM.pdf]

Supplemental Table 5. Quantitative data of phenotypes of WT, *25*, *346*, and *23456* mutants

**Phenotype**	**WT**	***25*** **double mutant**	***346*** **triple mutant**	***23456*** **quintuple mutant**
**Plant Height (cm)**	38.3 ± 2.0	38.0 ± 2.1	34.4 ± 1.9[a]	32.3 ± 3.7[c]
**Rosette Number**	26.2 ± 3.5	23.8 ± 3.6	25.8 ± 3.1	19.7 ± 4.3[c]
**Flowering Time (days)**	25.6 ± 0.9	26.1 ± 0.8	25.8 ± 0.9	27.6 ± 1.4[c]
**Siliques per plant**	278.1 ± 50.4	206.5 ± 43.6[b]	184.4 ± 31.6[c]	97.0 ± 58.7[c]
**Silique Length (mm)**	20.3 ± 3.5	19.7 ± 0.5	22.2 ± 2.7[c]	16.4 ± 3.8[c]
**Seeds per silique**	40.0 ± 7.6	41.8 ± 9.8	45.1 ± 6.6[c]	26.8 ± 9.8[c]

* Five-week-old plants were used for plant height and rosette number measurements, silique data were taken from six-week-old plants, and approximately 15-20 plants were used for each genotype (a, P<0.05; b, P<0.01; c, P<0.001).
