# Supplementary material for: CRISPR-Cas9 multiplex genome editing of the hydroxyproline-O-galactosyltransferase gene family alters arabinogalactan-protein glycosylation and function in Arabidopsis
Source: BMC Plant Biol. 2021 Jan 6;21:16. doi: 10.1186/s12870-020-02791-9 (PMC7789275; doi:10.1186/s12870-020-02791-9)
Supplement: Supplementary file 6 — Additional file 6: Supplemental Table 6. List of primers for sequencing off-targets of the GALT genes. [file 12870_2020_2791_MOESM6_ESM.pdf]

Supplemental Table 6. List of primers for sequencing off-targets of the *GALT* genes

Primer Name	Sequence
3-1_At2g34660_F	GTTTTGGTGGGGAGGATTTT
3-1_At2g34660_R	GTTTCCGAATCTGCCAAGAA
3-2_At1g70100_F	CAGAACCGGGAAAGAACAGA
3-2_At1g70100_R	TTACCGGTTTCTTCGTCACC
3-3_At2g13520_F	CCAAATTGGCCTAGAATTGC
3-3_At2g13520_R	CCTTTGCTTGAAAACCATTCA
4-1_At2g19130_F	GCTTGCCAAGGAGATTGTTC
4-1_At2g19130_R	TCAAGCCTCTTCACCGCTAT
6-1_At4g15545_F	CTAAGCCGACACCAAACGAT
6-1_At4g15545_R	TGTGGGCGTTAAGATCCTTC
