# Supplementary figures and images for: CRISPR-Cas9 multiplex genome editing of the hydroxyproline-O-galactosyltransferase gene family alters arabinogalactan-protein glycosylation and function in Arabidopsis
Source: BMC Plant Biol. 2021 Jan 6;21:16. doi: 10.1186/s12870-020-02791-9 (PMC7789275; doi:10.1186/s12870-020-02791-9)

Supplemental Figure 1-A

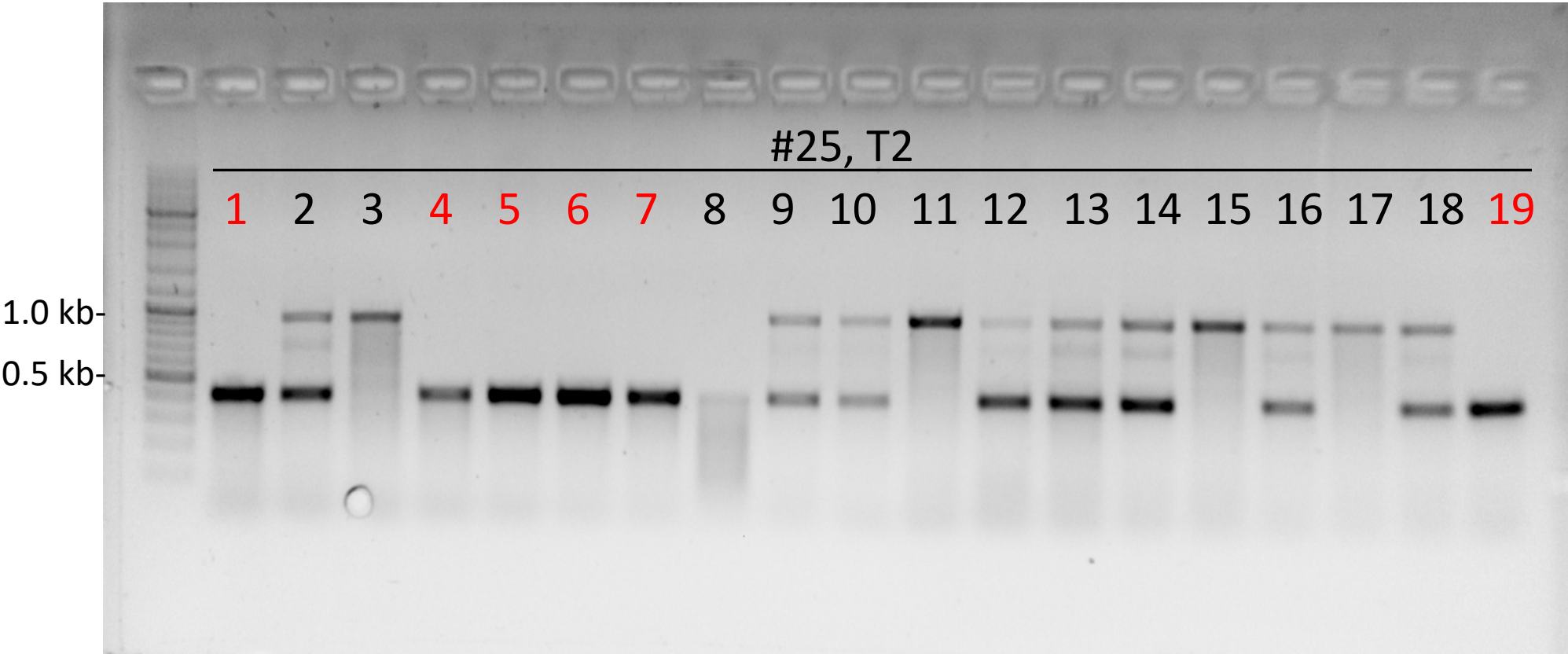

Supplemental Figure 1-B

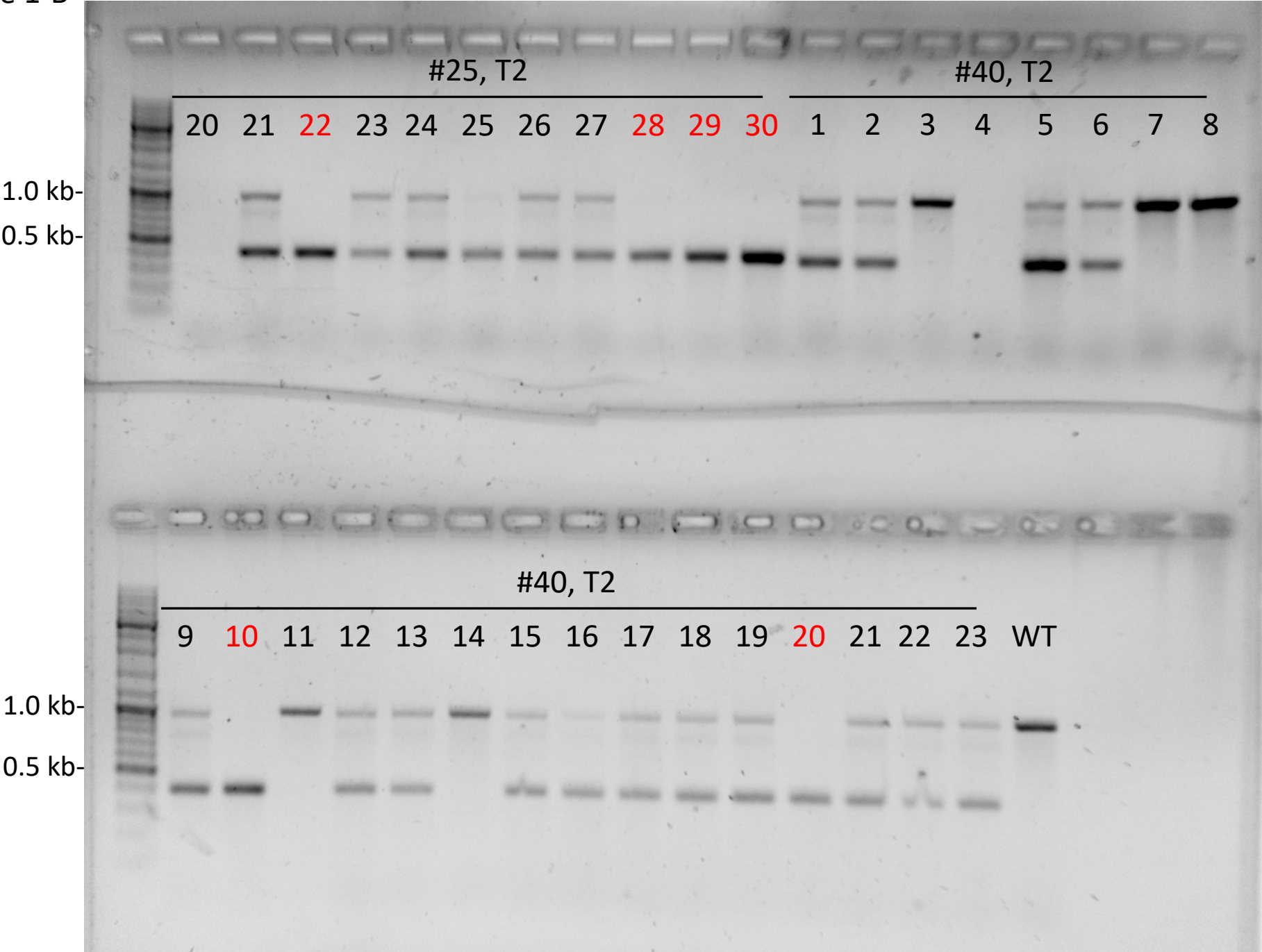

Supplemental Figure 1-C

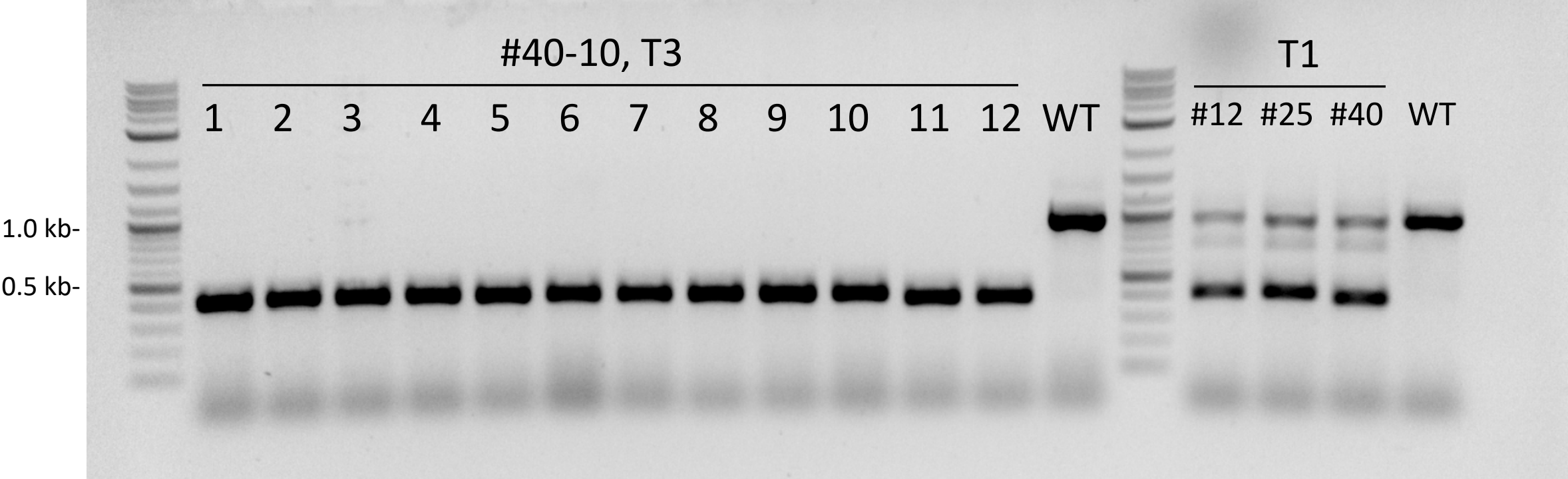

Supplemental Figure 2

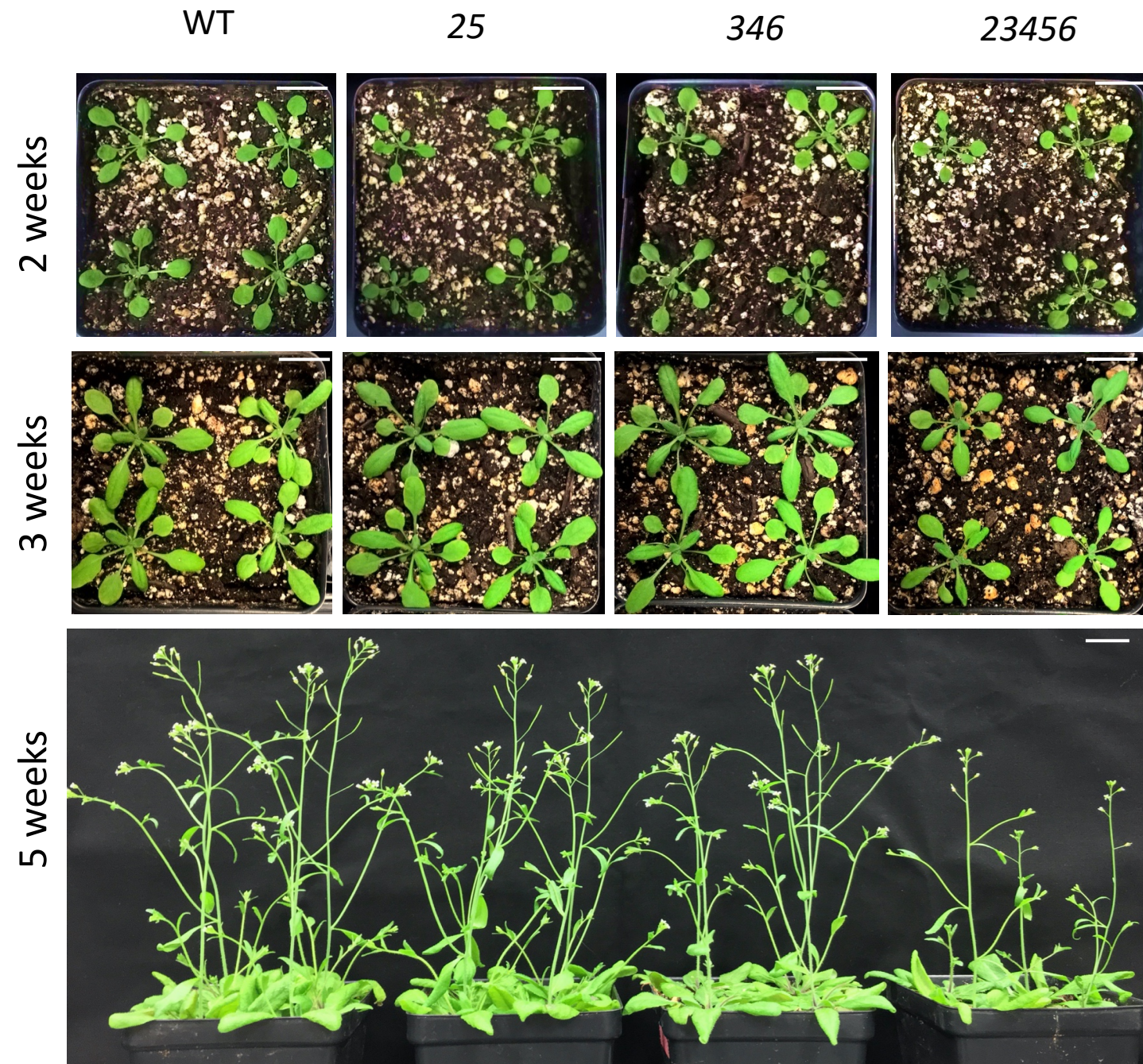

Supplement: Supplementary file 7 — Additional file 7: Supplemental Figure 1. Gene editing events of GALT3 generated by gRNA 3-1 and gRNA 3-2 were inherited to the next generation. (A-B) Segregation patterns of gene editing events of GALT3 in two transgenic lines (#25 and #40 ) in the T2 generation; (C) (Left panel) Gene deletion of GALT3 was inherited to the T3 generation in the #40-10 CRISPR line; (Right panel) Gene editing events of GALT3 were detected in three independent transgenic lines (#12, #25 and #40) in the T1 generation. Red indicated a full deletion in the GALT3 gene. Supplemental Figure 2. Growth phenotypes of the galt mutants in soil. Photographs of the galt mutants and WT were taken from the third week to the fifth week after sowing. Scale bar = 2 cm. [file 12870_2020_2791_MOESM7_ESM.pdf]
